# Lung function and cognitive ability in children: a UK birth cohort study

**DOI:** 10.1136/bmjresp-2022-001528

**Published:** 2023-05-02

**Authors:** Jack Grenville, Raquel Granell, James Dodd

**Affiliations:** 1 Respiratory Medicine, Cardiff and Vale University Health Board, Cardiff, Wales, UK; 2 MRC Integrative Epidemiology Unit, University of Bristol, Bristol, UK; 3 Academic Respiratory Unit, University of Bristol, Bristol, UK; 4 Respiratory Medicine, North Bristol NHS Trust, Bristol, UK

**Keywords:** Lung Physiology, Paediatric Lung Disaese, Psychology, Systemic disease and lungs, Respiratory Measurement

## Abstract

**Background:**

Decreased adult lung function is associated with subsequent impairment in cognition. A similar relationship in early life could be of great policy importance, since childhood cognitive ability determines key adult outcomes, including socioeconomic status and mortality. We aimed to expand the very limited data available on this relationship in children, and hypothesised that reduced lung function would be longitudinally associated with decreased cognitive ability.

**Methods:**

Lung function was measured at age 8 (forced expiratory volume in one second (FEV_1_), forced vital capacity (FVC); % predicted), and cognitive ability was measured at ages 8 (Wechsler Intelligence Scale for Children, third edition) and 15 (Wechsler Abbreviated Scale of Intelligence), in the Avon Longitudinal Study of Parents and Children. Potential confounders were identified as preterm birth, birth weight, breastfeeding duration, prenatal maternal smoking, childhood environmental tobacco smoke exposure, socioeconomic status and prenatal/childhood air pollution exposure. Univariable and multivariable linear models (n range=2332–6672) were fitted to assess the cross-sectional and longitudinal associations of lung function with cognitive ability, and change in cognitive ability between ages 8 and 15.

**Results:**

In univariate analyses, both FEV_1_ and FVC at age 8 were associated with cognitive ability at both ages, but after adjustment, only FVC was associated with full-scale IQ (FSIQ) at ages 8 (β=0.09 (95% CI 0.05 to 0.12; p<0.001)) and 15 (β=0.06 (0.03 to 0.10; p=0.001)). We did not find evidence of an association between either lung function parameter and interval change in standardised FSIQ.

**Discussion:**

Reduced FVC, but not FEV_1_, is independently associated with decreased cognitive ability in children. This low-magnitude association attenuates between ages 8 and 15, while no association is evident with longitudinal change in cognitive ability. Our results support a link between FVC and cognition across the life course, possibly due to shared genetic or environmental risk, rather than causation.

**WHAT IS ALREADY KNOWN ON THIS TOPIC**

- Reduced lung function has repeatedly been shown to share a longitudinal association with cognitive dysfunction in middle-aged and older adults, but little data exists on whether lung function and cognition might also be related in children.

**WHAT THIS STUDY ADDS**

- In only the second, and by far the largest, cohort study to examine this question in children, we find an independent association between forced vital capacity at age 8, and cognitive ability at ages 8 and 15.

**HOW THIS STUDY MIGHT AFFECT RESEARCH, PRACTICE OR POLICY**

- Our findings should prompt research into the possible causality of this association, which, if established, would reinforce the importance of enacting policy to protect children’s lung health.

## Introduction

Decreased lung function is associated with a range of adverse multisystem health outcomes, including impaired cognition.^1 2^ The recurrent observation of an independent, and temporally sequential, association between reduced lung function and cognition has led some to hypothesise that it might be causal.^3 4^ Due to the importance of childhood measurements of cognition in predicting adult socioeconomic and health outcomes,^5 6^ if they were shown to be causally affected by reduced childhood lung function, there would be profound implications for policy to protect children’s lung health. However, at present, only very limited research exists in this age group.^7^ Our study aims to investigate, using data from a large UK birth cohort, whether decreased lung function is longitudinally associated with lower cognitive ability in children.

Multiple studies, including a meta-analysis of 8 cohorts comprising 20 586 participants in Europe and North America,^2^ 2 US-based cohort studies of 14 184 participants^8^ and 1377 participants,^9^ and a Swedish-based twin study (n=832),^3^ have demonstrated an independent, longitudinal association between reduced lung function and subsequent impairment of cognition in older adults.

Studies in adults tend to measure cognition using clinical records of the development of pathological cognitive impairment or dementia, or cognitive testing to detect milder impairments. In children, it is typical to assess cognitive ability (also known as general intelligence or g), through tests of intelligence quotient (IQ). While IQ is not free from controversy as a measure of cognitive ability,^10^ childhood IQ scores are independently predictive of key adult life outcomes, including adult socioeconomic status (SES)^5^ and mid-to-late life mortality.^6^ The importance of investigating possible determinants of such a major contributor to children’s life chances should not be in doubt.

Only one study has specifically sought to investigate the relationship between lung function and cognitive ability in children.^7^ 165 children in Boston, USA, had lung function measured at age 6, and cognition measured at age 9, using the Kaufman Brief Intelligence Test (K-BIT) and the Wide Range Assessment of Memory and Learning (WRAML). Increases in forced expiratory volume in one second (FEV_1_), and forced vital capacity (FVC) (% predicted values for age, height, sex and ethnicity), were associated with higher composite scores on the K-BIT (β=0.23 (95% CI 0.07 to 0.39) and 0.18 (95% CI 0.03 to 0.33), respectively) and verbal, visual and learning subscales of the WRAML. Our study aims to expand the data addressing this research question with a larger sample from a UK birth cohort, and we hypothesise that a similar independent longitudinal association between lung function and cognitive ability will be observed.

## Methods

### Study participants

**Figure 1:**
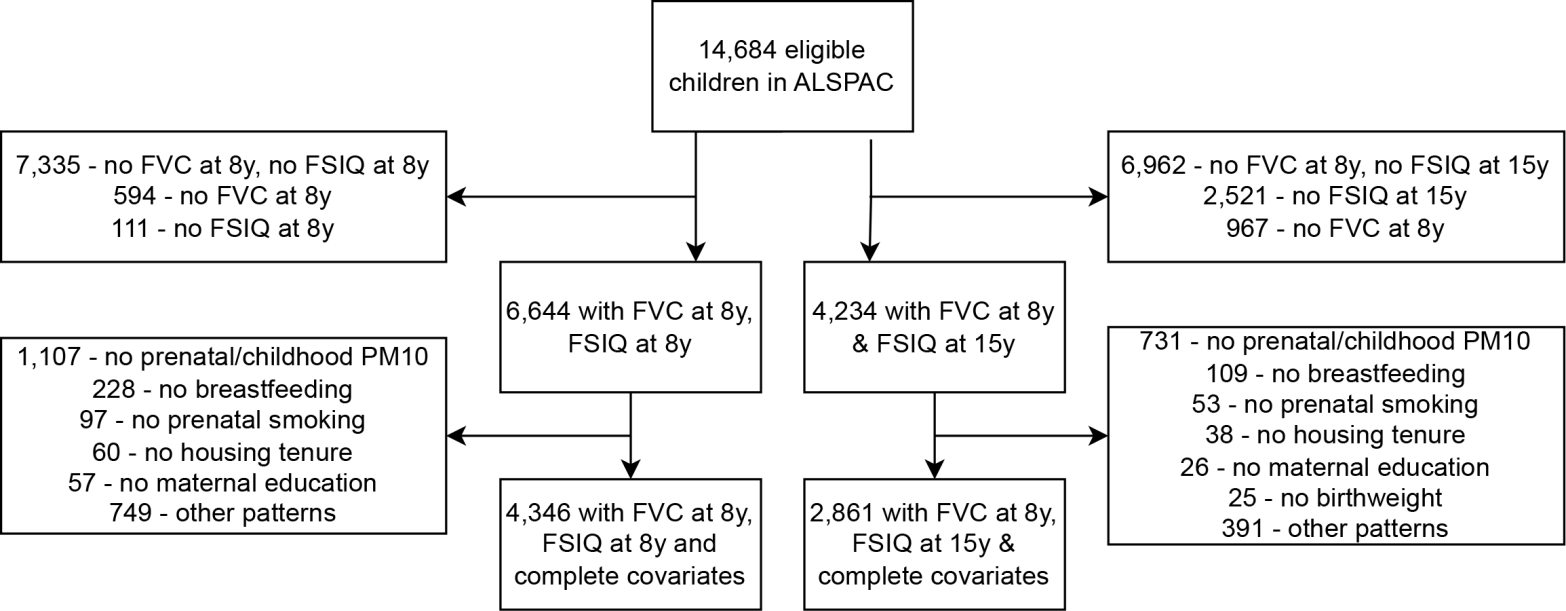
Flow chart showing study participants included in each analysis, and exclusions by missing variable. ALSPAC, Avon Longitudinal Study of Parents and Children; FSIQ, full-scale IQ; FVC, forced vital capacity; PM10, particulate matter less than 10 µm in diameter.

The Avon Longitudinal Study of Parents and Children (ALSPAC) is a prospective cross-generational cohort study based in Bristol, UK. A total of 14 541 pregnant women living in the former administrative county of Avon were recruited between 1 April 1991 and 31 December 1992. From those pregnancies initially recruited, 13 988 children were alive at 1 year, with a further 913 children born during the recruitment period later enrolled.^11 12^ Data on potential exposures and outcomes was collected through multiple questionnaires and clinic visits during pregnancy and childhood. Triplets and quadruplets were not included in our sample, and to reduce data clustering, twins were removed at random in relation to their birth order. A total of 14 684 children were therefore eligible for this study, with 6644 having lung function and cognitive ability data at age 8 and 4234 having exposure data at age 8 and outcome data at age 15 (figure 1). A full description of the study data, along with a searchable data dictionary and variable catalogue, is available on the study website.^13^

### Exposure

Lung function was measured using a hand-held spirometer (Vitalograph 2120; Vitalograph, UK), to American Thoracic Society standards, at age 8 (median 8.6 years; range 7.5–10.7), producing prebronchodilation measurements of FEV_1_, and FVC, in millilitres. Our exposure variables, expressed as percentages of values predicted for age, height, sex and ethnicity, were derived from equations from the Global Lung Function Initiative (GLI).^14^

### Outcome

Cognitive ability was measured using the Wechsler Intelligence Scale for Children—third Edition (WISC-III)^15^ at age 8, and the Wechsler Abbreviated Scale of Intelligence (WASI)^16^ at age 15 (median 15.4 years; range 14.3–17.7). The Wechsler scales have a long history, strong theoretical underpinnings, wide acceptance, and good external validation as tools for measuring cognitive ability in children.^10^

The WISC-III produces scores for full-scale IQ (FSIQ), verbal IQ (VIQ) and performance IQ (PIQ). VIQ relates to ‘crystallised intelligence’, which is context specific and dependent on prior education, while PIQ attempts to measure ‘fluid intelligence’, which involves visuospatial problem-solving, and is therefore less influenced by these factors.^17^ VIQ can be further subdivided into the Verbal Comprehension (VC) and Freedom From Distractibility (FD) Indices, which are derived from information, similarities, vocabulary, comprehension, digit span, and arithmetic tasks.^17^ PIQ can be subdivided into the Perceptual Organisation (PO) and Processing Speed (PS) Indices, which are compiled from picture completion and arrangement, block design, object assembly, coding, and symbol search tasks.^17^ PS is not recorded in the ALSPAC dataset, but VIQ, PIQ, VC, FD, and PO at age 8 were all included as outcomes in regression models, in an attempt to discern if lung function parameters share distinctive associations with specific facets of children’s cognition.

The WASI is an abbreviated version of the Wechsler Adult Intelligence Scale, and normally produces scores for FSIQ, VIQ derived from vocabulary and similarities subtests, and PIQ derived from matrix reasoning and block design subtests.^18^ However, for logistical reasons, and using a method validated by the test authors, ALSPAC participants completed only the vocabulary and matrix reasoning subtests at age 15. This produced FSIQ, but not VIQ or PIQ scores. This variation in methodology means that the FSIQ scores at ages 8 and 15 are not directly comparable. Therefore, we standardised these variables (y-mean/SD), before calculating interval change in SD from the mean FSIQ between ages 8 and 15.

### Covariates

**Figure 2:**
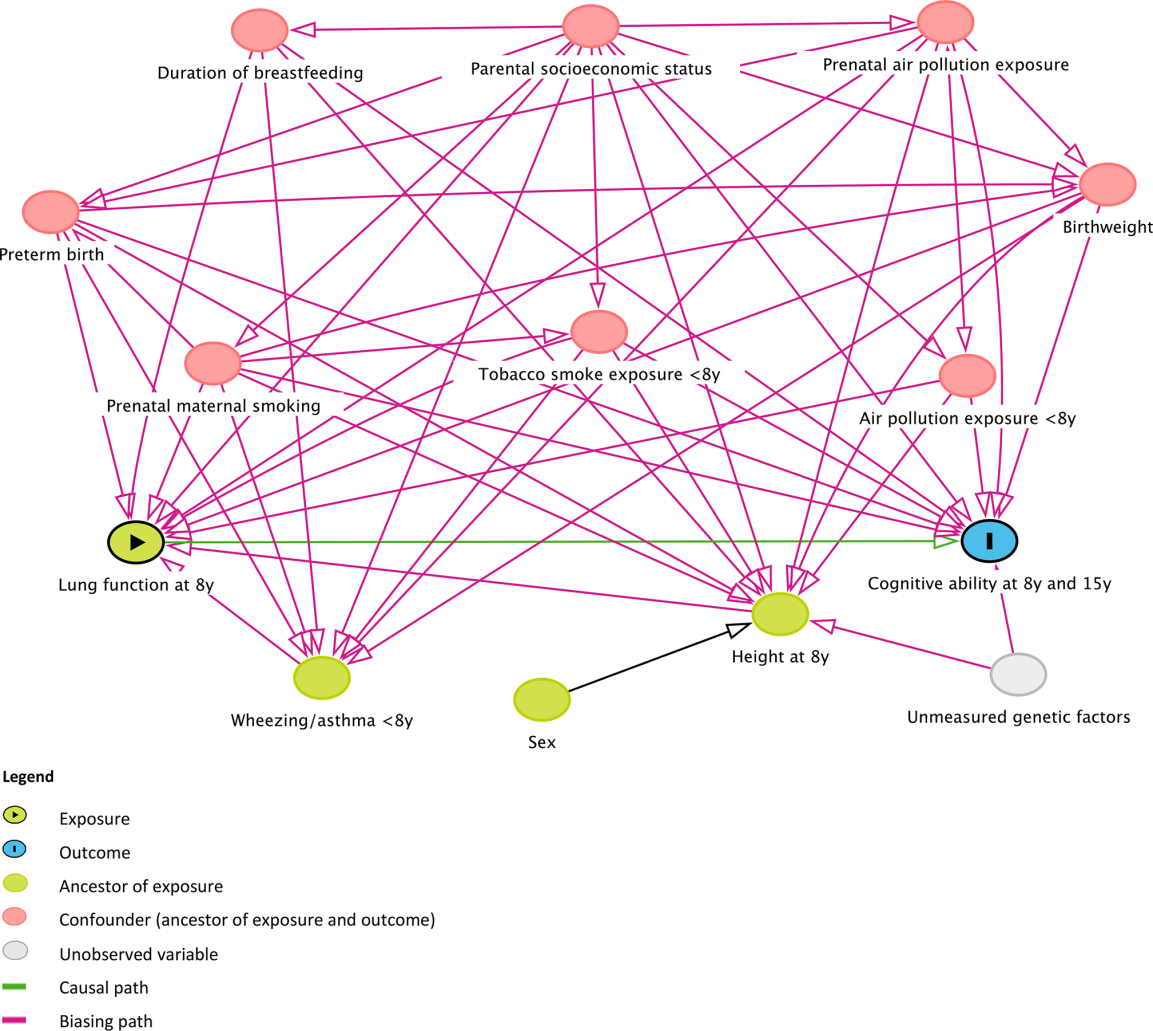
Directed acyclic graph codifying assumptions about relationships between exposure, outcome, and related variables. Diagram constructed using DAGitty - http://www.daggity.net.^19^

Potential confounders were identified through a combination of the authors’ prior subject knowledge, a systematic literature search for evidence on risk factors for exposure and outcome, and construction of a directed acyclic graph (DAG) (figure 2).^19^ We found evidence for a common effect on lung function and cognitive ability in childhood from parental SES,^20 21^ prenatal maternal smoking^22 23^ and air pollution exposure,^24 25^ preterm birth,^26 27^ birth weight,^22 28^ duration of breast feeding,^29 30^ childhood environmental tobacco smoke^31 32^ and air pollution exposure.^24 25^ The relationships identified by the DAG determined what would be adjusted for in our multivariable analysis. Age, height, sex and ethnicity were not included in our models due to the use of GLI % predicted values for lung function, which are preadjusted for these. Following suggestions from a reviewer, we conducted a sensitivity analysis to ensure that these variables had been adequately controlled for, by including them in the multivariable models estimating the effect of FEV_1_ and FVC at age 8 on FSIQ at ages 8 and 15, and on interval change in standardised FSIQ. We then included sex as an interaction term in these models, to investigate whether the effect of lung function parameters on FSIQ might differ between males and females. Those models that provided evidence of effect modification by sex were refitted separately for males and females.

Obstetric records provided data on birth weight (categorised into quintiles) and preterm birth (gestational age </> 37 weeks). Prenatal maternal smoking (yes/no) was measured by a maternal questionnaire for the first and second trimesters at 18 weeks’ gestation, and for the third trimester at 8 weeks postnatal. The duration of breast feeding (never/</> 6 months) was measured by questionnaire at 15 months of age. The child’s reported daily exposure to environmental tobacco smoke in hours was measured by a maternal questionnaires at 6, 15, 24, 38, 54, 65, 77 and 104 months of age. The mean reported value (disregarding missing values) was then categorised as ‘never’, less than, or greater than 1-hour mean reported daily exposure. Parental SES was measured by a maternal questionnaire using the proxy variables of maternal education (32 weeks’ gestation; ‘low’ if ‘O’ level or below), and housing tenure (6 weeks’ gestation; owner occupied or rented). Prenatal and childhood air pollution exposure were measured using data for exposure to particulate matter less than 10 µm in diameter (PM10; mean daily maternal exposure during pregnancy and cumulative childhood exposure to age 7) produced by Gulliver *et al*.^33^ Asthma diagnosis and childhood wheezing phenotype, which our DAG identified as potentially being on the causal pathway between exposure and outcome, were included in a separate multivariable model, in order to assess their contribution to any observed association (see online supplemental file 1).

10.1136/bmjresp-2022-001528.supp1 Supplementary data

### Statistical analysis

Cognitive test scores (FSIQ at ages 8 and 15, subscale scores at 8), FEV_1_ and FVC were treated as continuous variables and used to fit univariable and multivariable linear regression models. Linear regression assumptions were examined for these variables by visual inspection of scatter plots, histograms and Q–Q plots of model residuals, and by assessing if inclusion of polynomial terms improved model fit.

Continuous covariates (birth weight, PM10 exposure data) were categorised into quintiles because of failure to satisfy linearity assumptions. All hypothesised confounders were included *a priori* in the multivariable models. Univariable and multivariable linear models were fitted for participants with complete data for all covariates. The final sample in these models, depending on the precise combination of outcome and exposure, was between 3501 and 4362 for cognitive ability outcomes at age 8, and between 2782 and 2861 for the outcomes requiring FSIQ at age 15 (see table 1). For comparison, we also fitted univariable models for all participants with exposure and outcome, but not necessarily covariate data, at each age (see online supplemental file 1).

**Table 1:** Results of univariable and multivariable linear regression in complete cases

Outcome ↓ Exposure →	FEV_1_ (% predicted) at age 8	FVC (% predicted) at age 8
FSIQ (WASI) at age 15		
Participants, n	2814	2861
Mean difference* (95% CI; p)	0.04 (0.001 to 0.08; p=0.05)	0.09 (0.05 to 0.12; p<0.001)
Adjusted mean difference† (95% CI; p)	0.01 (–0.02 to 0.05; p=0.47)	0.06 (0.03 to 0.10; p=0.001)
Change in standardised FSIQ 8–15 years		
Participants, n	2782	2828
Mean difference‡ (95% CI; p)	−0.0004 (–0.003 to 0.002; p=0.76)	0.0001 (–0.003 to 0.003; p=0.99)
Adjusted mean difference† (95% CI; p)	−0.0004 (–0.003 to 0.002; p=0.73)	−0.00007 (–0.003 to 0.003; p=0.96)
FSIQ (WISC-III) at age 8		
Participants, n	4273	4346
Mean difference* (95% CI; p)	0.07 (0.03 to 0.11; p=0.001)	0.11 (0.07 to 0.15; p<0.001)
Adjusted mean difference† (95% CI; p)	0.03 (–0.01 to 0.07; p=0.10)	0.08 (0.05 to 0.12; p<0.001)
VIQ at age 8		
Participants, n	4289	4362
Mean difference§ (95% CI; p)	0.07 (0.03 to 0.11; p=0.001)	0.11 (0.07 to 0.15; p<0.001)
Adjusted mean difference† (95% CI; p)	0.03 (–0.01 to 0.07; p=0.09)	0.08 (0.04 to 0.12)
PIQ at age 8		
Participants, n	4286	4359
Mean difference§ (95% CI; p)	0.06 (0.02 to 0.10; p=0.005)	0.09 (0.05 to 0.14; p=0.005)
Adjusted mean difference† (95% CI; p)	0.03 (–0.01 to 0.07; p=0.12)	0.07 (0.03 to 0.11; p<0.001)
VC at age 8		
Participants, n	4261	4334
Mean difference§ (95% CI; p)	0.04 (0.02 to 0.07; p=0.002)	0.07 (0.04 to 0.10; p<0.001)
Adjusted mean difference† (95% CI; p)	0.02 (–0.01 to 0.04; p=0.18)	0.05 (0.03 to 0.08; p<0.001)
FD at age 8		
Participants, n	3501	3559
Mean difference§ (95% CI; p)	0.03 (0.02 to 0.05; p<0.001)	0.04 (0.03 to 0.05; p<0.001)
Adjusted mean difference† (95% CI; p)	0.02 (0.01 to 0.04; p=0.01)	0.03 (0.02 to 0.05; p<0.001)
PO at age 8		
Participants, n	4055	4126
Mean difference§ (95% CI; p)	0.03 (0.003 to 0.06; p=0.03)	0.05 (0.02 to 0.08; p<0.001)
Adjusted mean difference† (95% CI; p)	0.01 (–0.01 to 0.04; p=0.34)	0.04 (0.01 to 0.06; p=0.01)

All values to 2 decimal places, unless<0.005, then to 1 significant figure.

*Mean difference in IQ points per point increase in Global Lung Function Initiative (GLI) per cent predicted values of FEV_1_ and FVC.

†Adjusted for preterm birth, birth weight, breastfeeding duration, prenatal maternal smoking, childhood environmental tobacco smoke exposure, maternal education, housing tenure, prenatal and childhood particulate matter less than 10 µm in diameter air pollution exposure.

‡Mean difference in interval change in standardised FSIQ, measured in SD from the mean, per point increase in GLI per cent predicted values of FEV_1_ and FVC.

§Mean difference in WISC-III subscale score per point increase in per cent predicted values of FEV_1_ and FVC.

FD, Freedom from Distractibility Index; FEV_1_, forced expiratory volume in one second; FSIQ, full-scale IQ; FVC, forced vital capacity; PIQ, performance IQ; PO, Perceptual Organisation Index; VC, Verbal Comprehension Index; VIQ, verbal IQ; WASI, Wechsler Abbreviated Scale of Intelligence; WISC-III, Wechsler Scale of Intelligence for Children 3rd Edition.

Statistical models were fitted using Stata V.15.0 (StataCorp, Texas, USA).

### Participant and public involvement

Through the ALSPAC ethics and law committee, participants are involved in the ethical oversight of the study, and through the original cohort advisory panel, they regularly advise on appropriate and relevant use of the study data, as well as study design and methods. The ALSPAC executive provided approval for this study, but neither participants nor members of the public were involved in its conception or design. The manuscript will be available to participants and the public via an open-access online journal article.

## Results

Table 2 provides sociodemographic information on participants in the whole sample, and in the subsamples with FVC measured at age 8, cognitive ability at ages 8 and 15, and for those with complete covariate data. Those who attended clinic at ages 8 and 15 were increasingly female, white, living in owner-occupied accommodation, and had mothers who were educated to a higher level, compared with the original sample. The complete cases in our study had a still higher proportion of owner occupiers (88.9% vs 85.8% with only exposure at age 8 and outcome at age 15).

**Table 2:** Sociodemographic information and descriptive statistics for main exposures and outcomes in the original sample and subsamples

	Whole sample (n=14 684)	Individuals with data on FVC at 8, FSIQ at age 8 (n=6644)	Individuals with data on FVC and FSIQ at age 8 and complete covariates (n=4346)	Individuals with data on FVC at age 8, FSIQ at age 15 (n=4234)	Individuals with data on FVC at age 8, FSIQ at age 15 and complete covariates (n=2861)
Sex—% male	51.0	50.0	50.6	48.0	48.4
Ethnic group— % white	95.0	96.2	96.4	96.4	96.4
Housing tenure—% owned	73.3	83.3	86.8	85.8	88.9
Maternal education— % > ‘O’ level	35.4	43.9	42.7	48.8	47.7
FEV_1_ % predicted—mean (SD)	NA	99.4 (12.0)	99.5 (11.9)	99.4 (12.0)	99.4 (12.0)
FVC % predicted—mean (SD)	NA	99.2 (12.4)	99.1 (12.3)	99.1 (12.4)	99.0 (12.4)
FSIQ in clinic—mean (SD)	NA	104.3 (16.4)	104.5 (16.2)	95.1 (13.0)	94.7 (12.9)

All values to 1 decimal place.

FEV_1_, forced expiratory volume in one second; FSIQ, full-scale IQ; FVC, forced vital capacity; NA, not applicable.

**Figure 3:**
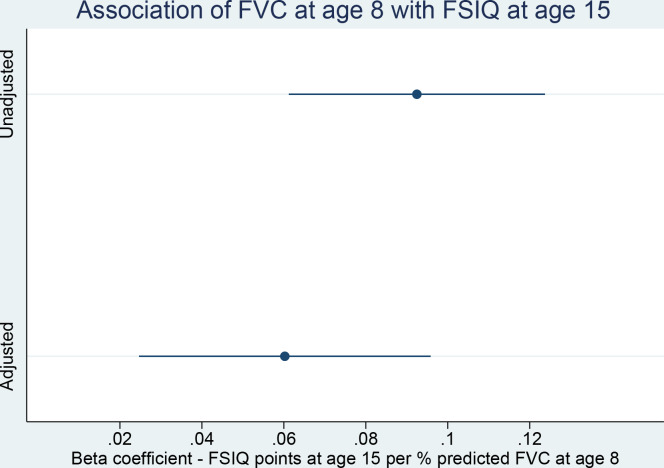
Association of forced vital capacity (FVC) at age 8 with full-scale IQ (FSIQ) at age 15.

Results of crude and adjusted linear regression models are shown in table 1. In the unadjusted analysis, we found that FSIQ at age 8 increased by a mean of 0.11 points (0.07–0.15; p<0.001), and FSIQ at age 15 by a mean of 0.09 (0.05, 0.12; p<0.001) points (figure 3), per percentage point increase in FVC at age 8. Beta coefficients for the association between FEV_1_ and FSIQ were 0.07 (0.03–0.11; p=0.001) at age 8, and 0.04 (0.001–0.08; p=0.05) at age 15. There was little evidence of heterogeneity of association of FEV_1_ or FVC with different subscale scores at age 8, with similar point estimates, and overlapping CIs, for VIQ and PIQ, as well as for the lower-order subscales of VC, PO and FD. We did not find evidence to suggest that differences in FEV_1_ or FVC were associated with change in standardised FSIQ between ages 8 and 15, with β=−0.0004 (−0.003 to 0.002; p=0.76), and 0.0001 (−0.003 to 0.003; p=0.99), respectively.

In the multivariable analysis, FSIQ at age 8 increased by a mean of 0.08 (0.05–0.12; p<0.001) points, and FSIQ at 15 by a mean of 0.06 (0.03–0.10; p=0.001) points (figure 3), for each 1% increase in FVC at age 8. However, little to no evidence remained of a cross-sectional or longitudinal association between FEV_1_ and FSIQ; β at age 8 was reduced to 0.03 (–0.01 to 0.07; p=0.10), and at 15 to 0.014 (–0.02 to 0.05; p=0.47). We again did not find evidence for a heterogenous association of FVC with subscale scores at age 8, or for an association of either lung function parameter with interval change in standardised FSIQ. Inclusion of sex, age and height at age 8 in our models yielded virtually identical results (see online supplemental file 1). When included as an interaction term in multivariable models, we found that sex, after also adjusting for age and height, did modify the effect of FEV_1_ on FSIQ at age 8 (p value for interaction=0.032), but not of FEV_1_ on FSIQ at age 15, nor of FVC on FSIQ at either age 8 or 15 (see online supplemental file 1). Separate multivariable analyses of the cross-sectional association between FEV_1_ and FSIQ at age 8 revealed β=0.07 (0.02–0.13; p=0.005) in girls, and β=−0.01 (−0.07 to 0.04; p=0.63) in boys (see online supplemental file 1). In our models including asthma and childhood wheezing as covariates, there was no attenuation of the association of FVC with cognitive ability (eg, for FSIQ at 15, β=0.06 (0.02–0.10); see online supplemental file 1).

## Discussion

### Summary of main findings

Our results reproduce the previously noted association between reduced FVC and lower cognitive ability in childhood. This was evident in the cross-sectional analysis at age 8, with an attenuated association persisting at age 15. Adjustment for hypothesised confounders resulted in a reduction of the estimated magnitude of the association of FVC with FSIQ at both 8 and 15 years of age, but there remained strong evidence for an association at both ages. We did not find evidence to suggest wide variations in the cross-sectional associations of FVC with the different elements of cognitive ability, as represented by the WISC-III subscale scores. While there was little evidence for an independent cross-sectional or longitudinal association between FEV_1_ and cognitive ability in the sample overall, we did find a cross-sectional association between FEV_1_ and FSIQ at age 8 in girls only. We failed to find evidence for an association of either FEV_1_ or FVC with longitudinal change in cognitive ability between ages 8 and 15. Inclusion in our models of asthma diagnosis and childhood wheezing as covariates did not attenuate the effect of FVC on cognition (see online supplemental file 1), suggesting that these two disorders do not contribute greatly to the observed effect, as might be expected, given that the bronchoconstriction characterising these conditions principally impairs FEV_1_.

### Summary of previous evidence

Our study replicates previous findings of an association between lung function and cognition, evidence for which has previously come mainly from middle-aged and older adults. One meta-analysis of 8 cohorts in the UK, Netherlands, Sweden and the USA, with a total of 20 586 participants (mean study recruitment age 65.6–82.8 years), examined associations between baseline measurements and longitudinal change in FEV_1_ and peak expiratory flow rate (PEFR), and multiple measures of cognitive performance over time.^2^ The authors found low-magnitude, but robust, associations between lower baseline measurements of, and rates of decline in, lung function parameters and scores on tests of multiple cognitive domains including mental status, PS, attention and working memory, perceptual reasoning, learning and memory, and verbal abilities.^2^ A large US-based cohort, following 14 184 participants (mean recruitment age 54.2 years) over 23 years, found increased odds of mild cognitive impairment or dementia with reduced baseline FEV_1_ (OR 1.11 (1.04–1.20) per SD decrease), FVC (OR 1.12 (1.05–1.20) per SD decrease) and obstructive (OR 1.33 (1.07–1.64)) or restrictive (OR 1.58 (1.14–2.19)) patterns of spirometry.^8^ Results from another US-based cohort of 1377 participants (mean recruitment age 79.4 years, 76% women) found that baseline reductions in a composite measure of lung function, derived from the average z-score of FEV_1_, FVC and PEFR, were longitudinally associated with more rapidly declining overall and domain-specific cognition.^9^ A study of 832 twins in Sweden (mean recruitment age 65.3 years), at 7 time points over 19 years, examined the question of directionality between lung function and cognition using dual change score models (DCSMs), and found evidence that declining FEV_1_ led to subsequent decrements in performance on assessments of spatial performance and PS.^3^ Those authors subsequently applied bivariate DCSMs to the same twin data, concluding that genetic influences on pulmonary function were the principal determinants of cognitive decline, rather than the observed relationship being explained by genetic or environmental confounding, or reverse causality.^4^ While cohort studies producing evidence of a longitudinal association between lung function and cognition in older adults are numerous, a systematic review of the literature in 2020 called into question the methodological quality of this evidence base, finding few studies meeting the inclusion criteria of having measured both variables on multiple occasions.^34^

Our study is only the second to find an association between lung function and cognition in children. The magnitude of our estimate of the association between FVC at age 8 and FSIQ at age 15 (0.06 (0.03–0.10)) is one-third that noted by Suglia *et al* between FVC at age 6 and IQ at age 9 (0.18 (0.03–0.33)),^7^ with a 20-point increase in FVC (% predicted) at age 8 in our cohort, conferring a 1.2-point difference in FSIQ at age 15. We did not replicate their finding of an independent effect of FEV_1_ on FSIQ in the sample overall, although an independent cross-sectional association was evident for girls at age 8 (see online supplemental file 1).

### Significance of main findings

Other authors have suggested the existence of a causal association between lung function and cognition across the life course,^3 4^ with possible mechanisms including intermittent cerebral hypoxia,^9 35^ nocturnal respiratory symptoms causing sleep disruption,^36^ or impairment of attention due to respiratory ill health.^37^ More recently, the proposed causality of this association has been thrown into doubt by a Mendelian Randomisation analysis, which suggested that previously noted associations may be due to residual confounding.^38^ While our methods do not permit strong statements about causality, it is possible to make inferences by observing the reduction in magnitude of the observed association between FVC and FSIQ between ages 8 and 15, and the lack of an association between FVC and longitudinal change in FSIQ. If the observed association between lung function and cognitive ability was indeed causal, it might be reasonable to postulate that those with lower lung function at age 8 would, over time, develop worsening cognition relative to peers, and that the association between FVC and FSIQ would not weaken over time, notwithstanding the introduction of other environmental effects on cognitive ability, or the possibility of selection bias due to participant dropout. Therefore, our results do not appear to support a causal link between lung function and cognitive ability between 8 and 15 years of age. They do not provide evidence for or against such a causal link earlier in life. However, rather than being causatively associated, it is also possible, and indeed may be more likely, that our findings reflect a pre-existing association arising from common genetic, prenatal, or early-life vulnerabilities. The independent association with cognitive ability found for FVC, but not FEV_1_, could be due to the fact that it is affected by a wider range of pathologies which might also impact childhood IQ, such as neurodevelopmental disorders and morbid obesity, or its greater heritability resulting in a higher proportion of genetic risk being shared with IQ (see online supplemental file 1 for further discussion).^39^

### Strengths and limitations

Our study has several strengths when compared with the solitary previous cohort study in children.^7^ The first of these is the much larger sample size permitted by the use of data from a pre-existing birth cohort, which likely resulted in the greater precision of our estimates. The second advantage of our study is that we selected covariates in a more systematic manner. The multivariable analysis in the aforementioned study adjusted for birth weight, gestational age, parental SES, prenatal maternal smoking, environmental tobacco smoke exposure, blood lead levels, childhood respiratory infection and asthma diagnosis. While the authors included a detailed rationale for the selection of each variable, we have conceptualised the relationships between our variables in a DAG, a more systematic approach that aims to avoid inappropriate inclusion of variables in the multivariable analysis, which may bias results. Indeed, in our DAG, we identified that childhood wheezing and asthma diagnosis might be on the causal pathway between exposure and outcome, meaning they were not adjusted for in the main analysis. A third advantage of our study, when compared with previous studies in both adults and children, is that cognitive ability was measured on more than one occasion, allowing for estimation of the association between lung function and longitudinal change in cognitive ability, and therefore inferences regarding the likelihood of a causal relationship.

Our study has a number of limitations. It is an observational study, and therefore it is quite possible that the observed association reflects residual confounding, selection or information bias, or reverse causality. It is probable that the confounding variables we have adjusted for in our multivariable analysis are not exhaustive, nor will their measurement have been without error. Even if of small magnitude for each variable, unmeasured confounding from omission of, or error in measuring, relevant factors may be a source of bias or loss of precision.

Table 2 illustrates that the missingness in our dataset is socially patterned, with participants of lower SES more likely to be absent from our sample, or to have missing data. While we have included correlates of social class as covariates, if there is a relationship between our outcomes and missingness in the dataset which is not accounted for by adjustment for these variables (either due to measurement error or unmeasured variability), our effect estimates will be subject to selection bias (see online supplemental file 1 for further details). Additionally, the generalisability of our findings is limited by the fact that our subsample is less representative of the population of Avon and the UK than the original ALSPAC sample, which itself had lower rates of households with single parents, who rented, had no car access, inhabited multiple-occupancy rooms, or were non-white, than the county and country at large.^11^ The ethnic homogeneity of the sample (96.2% white), in particular, means that our results can be considered of greatest relevance to Western European Caucasian populations.

In addition to these potential sources of bias, a further possible explanation of the observed association between FVC and cognitive ability might be the volitional nature of the measurement of FVC, which, unlike FEV_1_, is effort dependent. It is possible that those children with lower IQ might have had more difficulty in understanding the instructions for how to perform the procedure. This would result in lower FVC measurements in those with lower IQ, or in other words, reverse causality between outcome and exposure. This could explain the signals in our data against a causal relationship in the other direction, and the observation of an association with cognitive ability for FVC, but not FEV_1_.

### Directions for future research

Future research could interrogate the possibility of a causal effect of FVC on cognitive ability in children, by employing methods for causal inference such as Mendelian Randomisation, as was recently performed in adults.^38^ If no evidence for causality were forthcoming, examination of the genetic and epigenetic risk shared between lung function and cognition might help explain the association.

## Conclusion

In summary, our results support an independent association between childhood FVC and cognitive ability, while refuting such a relationship for FEV_1_. Between ages 8 and 15, the association of FVC with cognitive ability attenuates, and FVC is not associated with longitudinal change in cognitive ability. These findings may imply a non-causal association, which could instead be due to shared genetic or environmental vulnerabilities.

## Data Availability

Data may be obtained from a third party and are not publicly available. The informed consent obtained from Avon Longitudinal Study of Parents and Children (ALSPAC) participants does not allow the data to be made freely available through any third party maintained public repository. However, data used for this submission can be made available on request to the ALSPAC Executive. The ALSPAC data management plan describes in detail the policy regarding data sharing, which is through a system of managed open access. Full instructions for applying for data access can be found here: http://www.bristol.ac.uk/alspac/researchers/access/. The ALSPAC study website contains details of all the data that are available (http://www.bristol.ac.uk/alspac/researchers/our-data/).
