# Supplementary material for: Lung function and cognitive ability in children: a UK birth cohort study
Source: BMJ Open Respir Res. 2023 May 2;10(1):e001528. doi: 10.1136/bmjresp-2022-001528 (PMC10163472; doi:10.1136/bmjresp-2022-001528)
Supplement: Supplementary data [file bmjresp-2022-001528supp001.pdf]

Lung function and cognitive ability in children: A UK birth cohort study

ONLINE SUPPLEMENT

Supplementary methods

In order to ascertain whether or not GLI equations had adequately controlled for age, sex and height in our main multivariable models, we conducted a sensitivity analysis which adjusted for these variables, in addition to other hypothesised confounders, in those models estimating the association of FEV_1 and FVC at age 8 with FSIQ at ages 8 and 15, and with interval change in standardised FSIQ. In these models, we then included sex as an interaction term to assess for any modification of the association of lung function parameters with FSIQ, or change in FSIQ, by sex. For those models providing evidence of interaction, we conducted separate multivariable analyses for males and females.

To facilitate comparison of the crude effect estimate in the sample as a whole with those who had complete data, we fitted univariable linear models including all participants for the outcomes of FSIQ at 8 and 15, and change in standardised FSIQ between these ages. Sample size with this approach increased to between 6,548 and 6,672 for the outcome of FSIQ at age 8, and between 4,122 and 4,234 for outcomes requiring FSIQ at age 15.

We wished to attempt to estimate the contribution of asthma and childhood wheezing illness to any observed association of lung function with cognitive ability. We therefore fitted univariable models restricted to participants with data on asthma diagnosis and childhood wheezing phenotype in addition to other covariates, and added these variables to the multivariate models of the effect of lung function on FSIQ at ages 8 and 15, and change in standardised FSIQ. These models permitted sample sizes of 3,346 to 3,372 for the outcome of FSIQ at age 8, and 2,306 to 2,365 for outcomes requiring FSIQ at age 15.

Asthma diagnosis was measured using a combination of parental questionnaire at 91 months (which asked if the child had ever been diagnosed with asthma by a doctor), as well as symptoms of asthma and medication history in the clinic at 8 years of age. Childhood wheezing phenotypes have been described for the ALSPAC cohort, and describe the trajectory of a child's wheezing in terms of onset and persistence.[1] They can be characterised as transient early, prolonged early, intermediate onset, late onset, persistent, or never/infrequent.[1] Different phenotypes have differing strengths of association with atopy, asthma diagnosis, and reduced lung function; persistent, intermediate and late onset wheezing confer a greater risk.[1] The phenotypes used in our study were derived by Henderson *et al*. from maternal questionnaire data on wheezing symptoms collected at seven time points between 0 and 7 years of age, which was then used to categorise participants into one of the phenotype described above.[1] Wheezing phenotype was included in multivariate analyses as an unordered categorical variable.

Grenville J, *et al. BMJ Open Resp Res* 2023; 10:e001528. doi: 10.1136/bmjresp-2022-001528

Lung function and cognitive ability in children: A UK birth cohort study

To facilitate estimation of the likelihood of our results being subject to selection bias due to missing data, following a method outlined by Cornish *et al*,[2] we fitted logistic regression models with the outcome of being a complete case at age 8 and 15. These models included as covariates all variables in our main multivariable linear models.

Supplementary results

Supplementary table 1 shows the results of multivariable models including sex, age and height. Results changed very little with their addition, with identical point estimates for the effect of FEV_1 and FVC at age 8 on FSIQ at ages 8 and 15, with some minor changes to confidence intervals and p values. Point estimates for the effect of lung function parameters on interval change in standardised FSIQ differed slightly, but remained very close to zero, with very large p values.

Supplementary table 1. Results of multivariable linear regression models including sex, exact age and height at age 8

**Outcome↓ Exposure→**	**FEV_1 % predicted at age 8**	**FVC % predicted at age 8**
**FSIQ (WASI) at age 15**		
Number of participants	2,814	2,861
Adjusted mean difference* (95% CI; p)	0.01 (-0.02, 0.05; p=0.45)	0.06 (0.03, 0.10; p=0.001)
**Change in standardised FSIQ age 8 to 15**		
Number of participants	2,782	2,828
Adjusted mean difference** (95% CI; p)	-0.0003 (-0.003, 0.002; p=0.86)	0.0001 (-0.003, 0.003; p=0.94)
**FSIQ (WISC-III) at age 8**		
Number of participants	4,273	4,346
Adjusted mean difference* (95% CI; p)	0.03 (-0.01, 0.07; p=0.16)	0.08 (0.04, 0.11; p<0.001)

FEV_1 – Forced Expiratory Volume in 1 second; FVC – Forced Vital Capacity; FSIQ – Full-scale Intelligence Quotient (IQ); WASI – Wechsler Abbreviated Scale of Intelligence; WISC-III – Wechsler Scale of Intelligence for Children 3rd Edition
*Mean difference in IQ points per point increase in GLI per cent predicted values of FEV_1 and FVC
**Mean difference in interval change in FSIQ (standard deviations), per point increase in GLI per cent predicted values of FEV_1 and FVC
$^{\Phi}$Adjusted for sex, age, height, preterm birth, birth weight, breastfeeding duration, prenatal maternal smoking, childhood environmental tobacco smoke exposure, maternal education, housing tenure, prenatal and childhood PM10 air pollution exposure.
All values to 2 decimal places, unless <0.005, then to 1 significant figure.

Supplementary table 2 shows the p-values for interaction when sex was included as an interaction term to assess for any modification of the association between FEV_1 and FVC at age 8 and cognitive scores at ages 8 and 15, as well as interval change in standardised FSIQ. There was minimal evidence for any interaction, except in the association between FEV_1 at age 8 and FSIQ at age 8, where the p value for interaction was 0.03.

Grenville J, *et al. BMJ Open Resp Res* 2023; 10:e001528. doi: 10.1136/bmjresp-2022-001528

Supplementary table 2. P values for interaction of exposure-outcome association with sex

Outcome↓ Exposure→	FEV_1 % predicted at age 8	FVC % predicted at age 8
**FSIQ (WASI) at age 15**		
p value for interaction with sex	0.51	0.75
**Change in standardised FSIQ age 8 to 15**		
p value for interaction with sex	0.23	0.23
**FSIQ (WISC-III) at age 8**		
p value for interaction with sex	0.03	0.22
**VIQ at age 8**		
p value for interaction with sex	0.10	0.35
**PIQ at age 8**		
p value for interaction with sex	0.08	0.32
**VC at age 8**		
p value for interaction with sex	0.11	0.48
**FD at age 8**		
p value for interaction with sex	0.55	0.33
**PO at age 8**		
p value for interaction with sex	0.12	0.40

FEV_1 – Forced Expiratory Volume in 1 second; FSIQ – Full-scale Intelligence Quotient (IQ); WISC-III – Wechsler Scale of Intelligence for Children 3rd Edition

Supplementary table 3 shows the separate multivariable analyses we conducted to assess the cross-sectional association between FEV_1 and FSIQ at age 8 in males and in females. This shows that the data did demonstrate a cross-sectional association between FEV_1 and FSIQ at age 8 for females, with FSIQ increasing by 0.07 points (0.02, 0.13; p=0.005), for each one per cent increase in the GLI per cent predicted value of FEV_1. No such association was evident for males, with a point estimate close to zero, a confidence estimate crossing the null, and a large p value. Despite the interaction p values providing only weak evidence of interaction with the effect of FEV_1 on subscale scores at age 8, we also fitted separate models for males and females with these outcomes, in order to determine whether any particular facet of cognitive ability could explain this observation. For simplicity, we have not displayed these here, but the pattern was similar, with strong evidence for an association between FEV_1 at age 8 and all subscale scores in females, but not in males. No specific subscale score appeared to explain the association between FEV_1 and FSIQ in females at age 8 more than others. Given this isolated gender-specific cross-sectional association of FEV_1 with FSIQ, we ran models to include asthma diagnosis and childhood wheezing phenotype as covariates in females only, to see if these variables might explain it. Results changed very little, with β=0.09 (0.03, 0.16; p=0.006), indicating that these variables did not explain the association.

Supplementary table 3. Results of multivariable linear regression of FSIQ at age 8 with FEV_1 at age 8 in males and females

Outcome↓ Exposure→	FEV_1 % predicted at age 8
**FSIQ (WISC-III) at age 8 – males**	
Number of participants	2,166
Adjusted mean difference*ᶲ (95% CI; p)	-0.01 (-0.07, 0.04; p=0.63)
**FSIQ (WISC-III) at age 8 – females**	
Number of participants	2,107
Adjusted mean difference*ᶲ (95% CI; p)	0.07 (0.02, 0.13 ; p=0.005)

FEV_1 – Forced Expiratory Volume in 1 second; FSIQ – Full-scale Intelligence Quotient (IQ); WISC-III – Wechsler Scale of Intelligence for Children 3rd Edition

*Mean difference in IQ points per point increase in GLI per cent predicted values of FEV_1

ᶲAdjusted for age, height, preterm birth, birth weight, breastfeeding duration, prenatal maternal smoking, childhood environmental tobacco smoke exposure, maternal education, housing tenure, prenatal and childhood PM10 air pollution exposure.

All values to 2 decimal places, unless <0.005, then to 1 significant figure.

Supplementary table 4 shows the results of the univariable linear regression models for all participants with data for exposures and FSIQ outcomes, but not necessarily covariates. In this model, estimates for the associations of FEV_1 and FVC with FSIQ at ages 8 and 15 were slightly higher than in the main univariate analysis, which was restricted to participants with complete data (e.g. for FVC and FSIQ at 8 years $\beta = 0.13$ [0.10, 0.16; $p<0.001$], compared to 0.11 [0.07, 0.15; $p<0.001$] in the main analysis). As in the main analysis, there was little to no evidence of an association of either FEV_1 or FVC with change in standardised FSIQ between 8 and 15 years of age.

Supplementary table 4. Results of univariable (unadjusted) linear regression for the outcomes of full-scale intelligence quotient (FSIQ) and change in standardised FSIQ for all study participants

Outcome↓ Exposure→	FEV_1 % predicted at age 8	FVC % predicted at age 8
**FSIQ (WASI) at age 15**		
Number of participants	4,179	4,234
Mean difference* (95% CI; p)	0.05 (0.02, 0.08 p=0.002)	0.09 (0.06, 0.12; p<0.001)
**Change in standardised FSIQ age 8 to 15**		
Number of participants	4,122	4,175
Mean difference** (95% CI; p)	-0.001 (-0.003, 0.001 ; p=0.32)	-0.001 (-0.003,0.0009; p=0.27)
**FSIQ (WISC-III) at age 8**		
Number of participants	6,548	6,672
Mean difference* (95% CI; p)	0.09 (0.05, 0.12; p<0.001)	0.13 (0.10, 0.16; p<0.001)

FEV_1 – Forced Expiratory Volume in 1 second; FVC – Forced Vital Capacity; WASI – Wechsler Abbreviated Scale of Intelligence; WISC-III – Wechsler Scale of Intelligence for Children 3rd Edition

*Mean difference in IQ points per point increase in GLI per cent predicted values of FEV_1 and FVC

** Mean difference in interval change in standardised FSIQ, measured in standard deviations from the mean, per point increase in GLI per cent predicted values of FEV_1 and FVC

These models include all participants, regardless of whether complete covariate data were available.

All values to 2 decimal places, unless <0.005, then to 1 significant figure.

Supplementary table 5 shows the results of uni- and multivariable models fitted for participants with complete data for asthma diagnosis and childhood wheezing phenotype, in addition to all other covariates. In the unadjusted analysis, the magnitude of effect estimates for FEV_1 and FVC is only very marginally reduced compared to the main univariable analysis (e.g. for effect of FVC on FSIQ at 15 β = 0.08 [0.04, 0.12; p<0.001], compared to 0.09 [0.05, 0.12; p<0.001] in the main analysis). In this analysis, while there remains strong evidence for a cross-sectional association between FEV1 and FSIQ at age 8, the estimate for the association of FEV_1 at age 8 with FSIQ at age 15 is reduced such that the 95% confidence intervals crosses the null, suggesting little evidence of a relationship between these two variables in the unadjusted analysis of this subsample. As in the main multivariable analysis, there is little to no evidence of an independent association between FEV_1 at age 8 and FSIQ at ages 8 or 15, or between FEV_1 or FVC at age 8 and interval change in standardised FSIQ. Point estimates for the association of FVC with FSIQ at ages 8 and 15 are unchanged, with a slight widening of confidence intervals (e.g. for FVC and FSIQ at 15 β = 0.06 [0.02, 0.10; p=0.004], compared to 0.06 [0.03, 0.10; p=0.001] in the main analysis).

Supplementary table 5. Results of univariable (unadjusted) and multivariable linear regression for the outcomes of FSIQ at ages 8 and 15, and change in standardised FSIQ, for participants with complete covariates, including asthma diagnosis and childhood wheezing phenotype

**Outcome↓ Exposure→**	**FEV_1 % predicted at age 8**	**FVC % predicted at age 8**
**FSIQ (WASI) at age 15**		
Number of participants	2,332	2,365
Mean difference* (95% CI; p)	0.04 (-0.002, 0.09; p=0.06)	0.08 (0.04, 0.12; p<0.001)
Adjusted mean difference$^{\Phi}$ (95% CI; p)	0.02 (-0.02, 0.06; p=0.38)	0.06 (0.02, 0.10; p=0.004)
**Change in standardised FSIQ age 8 to 15**		
Number of participants	2,306	2,342
Mean difference** (95% CI; p)	-0.001 (-0.004, 0.002; p=0.50)	-0.0002 (-0.003, 0.003; p= 0.86)
Adjusted mean difference$^{\Phi}$ (95% CI; p)	-0.0007 (-0.004,0.002; p=0.62)	-0.0003 (-0.003, 0.002; p= 0.81)
**FSIQ (WISC-III) at age 8**		
Number of participants	3,346	3,372
Mean difference* (95% CI; p)	0.08 (0.03, 0.12; p=0.001)	0.11 (0.06, 0.15; p<0.001)
Adjusted mean difference$^{\Phi}$ (95% CI; p)	0.03 (-0.01, 0.07; p=0.16)	0.08 (0.04, 0.12; p<0.001)

FEV_1 – Forced Expiratory Volume in 1 second; FVC – Forced Vital Capacity; WASI – Wechsler Abbreviated Scale of Intelligence; WISC-III – Wechsler Scale of Intelligence for Children 3rd Edition
*Mean difference in IQ points per point increase in GLI per cent predicted values of FEV_1 and FVC
** Mean difference in interval change in standardised FSIQ, measured in standard deviations from the mean, per point increase in GLI per cent predicted values of FEV1 and FVC
$^{\Phi}$After adjustment for preterm birth, birth weight, breastfeeding duration, prenatal maternal smoking, childhood environmental tobacco smoke exposure, maternal education, housing tenure, prenatal maternal and childhood PM10 air pollution exposure, asthma diagnosis and childhood wheezing phenotype.
All values to 2 decimal places, unless <0.005, then to 1 significant figure.

Supplementary table 6 summarises the results of logistic regression models which were fitted to identify determinants of being a complete case, and therefore of selection, at ages 8 and 15. After adjustment for all other covariates included in our original linear models, the odds of selection were reduced by having a mother educated to the equivalent of ‘O’ level or below at both ages 8 (Odds ratio [OR] 0.76 [0.60, 0.97; p=0.03]) and 15 (OR 0.58 [0.47, 0.72; p<0.001]). Meanwhile, those with higher FSIQ scores were increasingly likely to be included at ages 8 (OR 1.01 [1.00, 1.02; p=0.003]) and 15 (OR 1.01 [1.01, 1.02; p<0.001]).

Supplementary table 6. Results of logistic regression to identify determinants of selection at ages 8 and 15

	Odds ratio – complete case at 8y	Odds ratio – complete case at 15y
**Maternal education ≤‘O’ level**	0.76 (0.60, 0.97; p=0.03)	0.58 (0.47, 0.72; p<0.001)
**FSIQ (WISC-III) at 8y**	1.01 (1.00, 1.02; p=0.003)	1.02 (1.01, 1.02; p<0.001)
**FSIQ (WASI) at 15y**	N/A	1.01 (1.01, 1.02; p=0.001)

Coefficients are shown only for variables which were identified as determinants of being a complete case, and therefore of selection, after adjustment for all covariates included in original linear models. Coefficients represent odds ratios for the likelihood of selection at ages 8 and 15. For FSIQ at ages 8 and 15, these are the odds ratios per point increase in FSIQ. All values to 2 decimal places, unless <0.005, then to 1 significant figure (95% CI; p value). N/A – not applicable.

Supplementary discussion

Inclusion of age, height and sex in our multivariable models resulted in near identical results for the estimates of the association of FEV_1 and FVC at age 8 with FSIQ at age 8 and 15, and very minimal change in the estimates of the association with interval change in standardised FSIQ. These results indicate that these variables had indeed been adequately controlled for in the original analysis.

When we investigated for interaction of the association between exposure and outcome with sex in the multivariable regression models, we found that while there was strong evidence of a cross-sectional association between FEV_1 and FSIQ at age 8 in females, in males there was none. This sex-specific association was not explained by asthma diagnosis or childhood wheezing phenotype. The association was not explained by any specific facet of cognitive ability, as measured by the WISC-III subscale scores. It is difficult to explain why decreased FEV_1 should be cross-sectionally associated with decreased FSIQ in girls, but not boys, at age 8. FEV_1 is an effort independent measurement, meaning it is very unlikely that this association can be explained by differences between males and females in the ability to perform the procedure correctly. The association was not explained by asthma diagnosis or wheezing phenotype, despite the fact that these were associated with reduced FSIQ in separate univariate modelling. Both asthma and pathological wheezing phenotypes were more prevalent in males than females (eg 17.6% of boys at age 8 had a diagnosis of asthma, versus 12.7% of girls), so if this were the explanation for the sex-specific association it would be expected

that the association would be stronger in boys. The sex-specific association for FEV_1 with FSIQ did not remain evident at age 15. Overall, the significance of this finding is unclear.

Our univariable models including all participants with data for exposure and outcome, but not necessarily covariates, had slightly larger coefficients than the main univariable analysis, and narrower confidence intervals. Although a larger sample size confers greater precision, it is likely that these effect estimates are inflated by confounding, for which it is not possible to control in the additional individuals included, due to a lack of covariate data. On the other hand, the fact that the coefficients are larger in this more unselected group, than in the main univariable analysis, may also support the notion that any selection bias, from analysing an increasingly select and affluent subsample, is likely to be towards the null. This is discussed further below.

Estimated coefficients for the association of FVC with FSIQ were only very slightly attenuated by inclusion of asthma and wheezing phenotype as covariates. This indicates that these diagnoses do not account for much of the variance in IQ explained by FVC. The notion that asthma and wheezing are important in explaining the relationship between FVC and FSIQ is also contested by a loss of precision, as demonstrated by the widening of confidence intervals in the estimates from models including these variables (although this might also be due to a smaller sample size). Another explanation for the attenuation of the coefficients in these models might be the aforementioned effect of selection bias towards the null, due to a more select subsample. The evidence from our results against asthma and wheezing being important explanators of the relationship between FVC and FSIQ is unsurprising, because FEV_1 is a better measure of the airflow obstruction which characterises asthma and bronchospasm.

It is useful to highlight the clinical differences between FEV_1 and FVC to potentiate the formulation of hypotheses as to why we have detected an effect on childhood cognition from the latter but not the former. West conceptualises the lungs and thorax as an air pump, the function of which is dependent on stroke volume, resistance of the airways, and the force applied to the piston.[3] The latter is unimportant in forced expiration, due to dynamic compression of the airways, which means that flow is independent of effort.[3] FEV_1 is the metric of airways resistance, which is affected by pathological processes which cause their narrowing or premature collapse.[3] The most common of these are asthma, which causes reversible bronchoconstriction due to hyperresponsiveness and inflammation of the airways, and chronic obstructive pulmonary disease (COPD), which causes airflow limitation due to chronically inflamed and narrowed airways (chronic bronchitis), and premature collapse due to the loss of radial traction caused by destruction of lung parenchyma distal to the terminal bronchiole (emphysema).[3] FVC is a measure of the 'stroke volume'

of the air pump, which is determined by the capacity for the thoracic cage, respiratory muscles, pleura and lung parenchyma to fill the lungs with air, and then expel it, through alterations in intrathoracic pressure.[3] This can be negatively impacted by diseases of the thoracic cage, such as kyphoscoliosis, neuromuscular disorders such as muscular dystrophy, diseases of the lung parenchyma such as pulmonary and cystic fibrosis, and diseases of the pleura such as effusion or pneumothorax. A ratio of the FEV_1 to FVC is often used to distinguish 'obstructive' from 'restrictive' patterns of spirometry, with the former showing a ratio of less than 0.8 due to airflow limitation (FVC may be normal or reduced, while total lung capacity measured by helium dilution may be increased), and the latter showing a normal or increased ratio, but in the presence of reduced FVC and total lung capacity.[3]

We have noted an effect on childhood cognition from FVC, but not FEV_1. However, as discussed elsewhere, our results do not appear to support causality. The most plausible explanation for the association may therefore be genetic and/or early developmental vulnerabilities shared between lung function and cognitive ability. Accordingly, it seems reasonable to suggest that the reason for FVC, but not FEV_1, being associated with cognitive ability is that it may be a better correlate of respiratory and overall health in early life. As discussed above, FVC measures respiratory 'pump function', and this is affected by a wider range of pathology than is FEV_1. Airflow limitation typically develops over a number of years in young asthmatic children and over decades in adults with COPD, whereas many of the disorders predominantly affecting FVC occur earlier in life. An example would be cerebral palsy, a developmental neurological disorder which can affect FVC and IQ. This and other early life neurodevelopmental disorders with a common effect on the two traits, which have not been controlled for in our analysis, could explain a proportion of the observed effect. Another consideration is the increasing problem of childhood obesity,[4] because morbid obesity can cause a restrictive lung function defect with reduced FVC. Elevated body mass index (BMI) has been associated with reduced IQ,[5] although it is not clear if this is a causal relationship. BMI was not controlled for in the multivariable analysis, so it is possible that part of the observed association of FVC with FSIQ is explained by obesity. Estimates of the heritability of pulmonary function traits vary widely, but FVC has generally been assessed to be slightly more heritable than FEV_1 or FEV_1/FVC.[6] Another explanation for the observed association, for FVC but not FEV_1, is that FVC might share more genetic architecture in common with cognitive ability than FEV_1, with its slightly higher heritability potentially making this more likely.

Selection bias, due to study attrition and missing data, is an important source of bias in statistical estimates of relationships between variables derived from cohort studies. We conducted a complete-case analysis, which for an unbiased effect estimate, requires that data is either missing completely at random (MCAR i.e. no systematic missingness which could induce bias), or missing at random (MAR), i.e. missing dependent on

the outcome or exposure only via measured covariates. Given the social patterning of missingness in the ALSPAC dataset demonstrated in table 1 and discussed in the main text, it is safe to assume that the MCAR assumption is not satisfied. We have controlled for SES, but this was imperfectly measured by two proxy variables (maternal education and housing tenure). It is probable that a failure to control for the unmeasured component of SES, as well as for other unmeasured factors related to both outcome and selection, mean that the MAR assumption is also unsatisfied. Cornish *et al*. showed that FSIQ at age 15 in ALSPAC is likely to be missing not at random (MNAR) i.e. missing dependent on its own (unknown) value, with those with higher IQs more likely to remain in the sample.[2] These findings are corroborated by the results of the logistic regression models we fitted using their method, to identify determinants of selection, which showed that not only is low maternal education negatively associated with inclusion, but that the odds of selection at each age are higher with increasing FSIQ scores, after adjustment for all other covariates. While it is not possible to demonstrate conclusively from the original dataset, these findings are strongly supportive of our outcomes being MNAR, a source of selection bias for which it is not possible to fully correct. According to the analysis of Cornish *et al*,[2] selection bias might have been reduced, and precision increased, by the use of educational data to impute missing values. Due to time constraints relating to thesis submission deadlines, we did not apply for educational records via data linkage for the purpose of multiple imputation (MI), meaning that there may be greater bias in our estimate than if we had performed MI. However, due to the likelihood of the outcome being MNAR, bias would not have been completely eliminated by MI, and it seems reasonable to assume that any selection bias affecting our estimates would be towards the null. This is because if proportionately more participants with comparatively lower IQ and lung function are lost to follow-up (which seems plausible due to the socially patterned nature of missingness), without completely adjusting for the cause of their deselection, then the observed association would be weakened. There is some evidence for the assertion that selection bias due to missing data is toward the null, from the fact that coefficients from univariable analysis limited to participants with complete data were of smaller magnitude than those from analyses including the less selected group of participants with exposure and outcome, but not necessarily covariate, data.

Supplementary references

1. Henderson J, Granell R, Heron J, et al. Associations of wheezing phenotypes in the first 6 years of life with atopy, lung function and airway responsiveness in mid-childhood. *Thorax* 2008;63(11):974-80.
2. Cornish RP, Tilling K, Boyd A, et al. Using linked educational attainment data to reduce bias due to missing outcome data in estimates of the association between the duration of breastfeeding and IQ at 15 years. *International Journal of Epidemiology* 2015;44(3):937-45. doi: 10.1093/ije/dyv035

3. West JB, Luks AM. West's pulmonary pathophysiology: the essentials: Lippincott Williams & Wilkins 2021.

4. Ng M, Fleming T, Robinson M, et al. Global, regional, and national prevalence of overweight and obesity in children and adults during 1980-2013: a systematic analysis for the Global Burden of Disease Study 2013. *The Lancet* 2014;384(9945):766-81. doi: 10.1016/S0140-6736(14)60460-8

5. Jacob L, Haro JM, Smith L, et al. Association between intelligence quotient and obesity in England. *Lifestyle Medicine* 2020;1(2):e11. doi: https://doi.org/10.1002/lim2.11

6. Williams PT. Spirometric traits show quantile-dependent heritability, which may contribute to their gene-environment interactions with smoking and pollution. *PeerJ* 2020;8:e9145-e45. doi: 10.7717/peerj.9145
